# Validation of the REM behaviour disorder phenoconversion-related pattern in an independent cohort

**DOI:** 10.1007/s10072-023-06829-2

**Published:** 2023-05-04

**Authors:** Beatrice Orso, Pietro Mattioli, Eun-Jin Yoon, Yu Kyeong Kim, Heejung Kim, Jung Hwan Shin, Ryul Kim, Claudio Liguori, Francesco Famà, Andrea Donniaquio, Federico Massa, David Vállez García, Sanne K. Meles, Klaus L. Leenders, Agostino Chiaravalloti, Matteo Pardini, Matteo Bauckneht, Silvia Morbelli, Flavio Nobili, Jee-Young Lee, Dario Arnaldi

**Affiliations:** 1 Department of Neuroscience, Rehabilitation, Ophthalmology, Genetics, Maternal and Child Health (DINOGMI), Clinical Neurology, University of Genoa, Largo Daneo 3, 16132 Genoa, Italy; 2 Memory Network Medical Research Center, Seoul National University, Seoul, South Korea; 3 Department of Nuclear Medicine, Seoul National University College of Medicine and Seoul Metropolitan Government-Seoul National University Boramae Medical Center, Seoul, South Korea; 4 Department of Neurology, Seoul National University College of Medicine, Seoul, South Korea; 5 Department of Neurology, Inha University Hospital, Incheon, South Korea; 6 Department of Systems Medicine, University of Rome ‘Tor Vergata”, Rome, Italy; 7 Sleep Medicine Center, Neurology Unit, University Hospital “Tor Vergata”, Rome, Italy; 8 IRCCS Ospedale Policlinico S. Martino, Largo Rosanna Benzi 10, 16132 Genoa, Italy; 9 Department of Radiology and Nuclear Medicine, Amsterdam UMC, Location VuMC, Amsterdam Neuroscience, Amsterdam, the Netherlands; 10 Department of Neurology, University of Groningen, University Medical Center Groningen, Groningen, The Netherlands; 11 Department of Nuclear Medicine and Molecular Imaging, University of Groningen, University Medical Center Groningen, Groningen, The Netherlands; 12 IRCCS Neuromed, Pozzilli, Italy; 13 Department of Biomedicine and Prevention, University of Rome “Tor Vergata”, Rome, Italy; 14 Department of Health Science (DISSAL), University of Genoa, Via Antonio Pastore 1, 16132 Genoa, Italy; 15 Department of Neurology, Seoul National University College of Medicine and Seoul Metropolitan Government-Seoul National University Boramae Medical Center, Seoul, South Korea

**Keywords:** REM behaviour disorder, [^18^F]FDG-PET, Alpha-synucleinopathies, Brain metabolic pattern, Parkinson’s disease, Dementia with Lewy bodies

## Abstract

**Background:**

A brain glucose metabolism pattern related to phenoconversion in patients with idiopathic/isolated REM sleep behaviour disorder (iRBDconvRP) was recently identified. However, the validation of the iRBDconvRP in an external, independent group of iRBD patients is needed to verify the reproducibility of such pattern, so to increase its importance in clinical and research settings. The aim of this work was to validate the iRBDconvRP in an independent group of iRBD patients.

**Methods:**

Forty iRBD patients (70 ± 5.59 years, 19 females) underwent brain [^18^F]FDG-PET in Seoul National University. Thirteen patients phenoconverted at follow-up (7 Parkinson disease, 5 Dementia with Lewy bodies, 1 Multiple system atrophy; follow-up time 35 ± 20.56 months) and 27 patients were still free from parkinsonism/dementia after 62 ± 29.49 months from baseline. We applied the previously identified iRBDconvRP to validate its phenoconversion prediction power.

**Results:**

The iRBDconvRP significantly discriminated converters from non-converters iRBD patients (*p* = 0.016; Area under the Curve 0.74, Sensitivity 0.69, Specificity 0.78), and it significantly predicted phenoconversion (Hazard ratio 4.26, C.I.95%: 1.18–15.39).

**Conclusions:**

The iRBDconvRP confirmed its robustness in predicting phenoconversion in an independent group of iRBD patients, suggesting its potential role as a stratification biomarker for disease-modifying trials.

## Introduction

Rapid eye movement (REM) sleep behaviour disorder (RBD) is a parasomnia presenting with dream-enacting behaviours resulting from the loss of the physiological muscle atonia during REM sleep [1].

RBD is usually defined as “idiopathic/isolated” (iRBD) when it is not associated by overt neurologic symptoms. Nonetheless, at least 70% of iRBD patients will eventually develop parkinsonism and/or dementia during follow-up [2-5]. However, phenoconversion time is highly variable, with patients not being phenoconverted after 10 years from diagnosis [6, 7]. Synucleinopathies have a long prodromal phase, and the presence of RBD is by far the strongest biomarker of an incipient neurodegenerative disease. Therefore, iRBD patients are good candidates for future disease-modifying therapies. However, efficient biomarkers able to predict short-term phenoconversion in iRBD patients are yet to be determined. [^18^F]fluorodeoxyglucose positron emission tomography ([^18^F]FDG-PET) is a promising biomarker of short-term phenoconversion. In fact, [^18^F]FDG-PET has been able to describe disease-specific brain metabolic patterns in several neurological conditions, including iRBD [8] and Parkinson’s disease (PD) [8, 9], thanks to the use of the Scaled Subprofile Modelling and Principal Component Analysis (SSM-PCA)[10]. This approach allows to extract covariance pattern between voxels.

In iRBD patients, the PD-related pattern (PDRP) expression increases over time [10], while the iRBD related pattern (RBDRP) expression decreases over time [11], suggesting that iRBD patients are changing their brain glucose metabolism pattern getting closer to the phenoconversion. Indeed, the RBDRP was identified in different cohort of patients [9, 11, 12], showing similar but not fully overlapping patterns. These data confirm that the brain glucose metabolism in iRBD patients is highly heterogenous [13], likely due to the different outcome phenoconversion diagnosis (i.e. parkinsonism-first versus dementia-first) and the phenoconversion timing (i.e. early-converters versus late/non-converters). As a further confirmation of the brain glucose metabolism heterogeneity in iRBD patients, it has been shown that the PDRP is a good predictor of phenoconversion, but the pattern obtained in de novo PD patients with RBD (dnPDRBD-RP) is even better [14]. However, it must be highlighted that iRBD patients could develop PD, dementia with Lewy bodies (DLB), and, less frequently, multiple system atrophy (MSA). Thus, a disease-specific related pattern, such as PDRP, or even dnPDRBD-RP, may not be the best choice for covering all the possible outcome.

Recently, a pattern of brain glucose metabolism associated with phenoconversion in iRBD (iRBDconvRP) has been demonstrated to be robust and reliable for discriminating iRBD converters from non-converters patients, and to have good predictive power, regardless of the phenoconversion diagnosis [15]. However, to confirm its clinical and research usefulness, the identified iRBDconvRP must be validated in an independent group of patients.

Therefore, the aim of the current study was to validate the phenoconversion prediction power of the previously described iRBDconvRP by applying it to a separate cohort of iRBD patients recruited from a different site.

## Materials and methods

### Patients

Forty iRBD patients (mean age 70 ± 5.59 years, 21 males) were enrolled between 2013 and 2015 from the Seoul National University College of Medicine (South Korea) [16, 17]. Diagnosis of RBD was confirmed by video-polysomnography, according to current criteria [18]. The exclusion criteria were set to rule out secondary causes for RBD, as previously described [16, 17]. Exclusion criteria were psychiatric or neurologic comorbidity, moderate to severe obstructive sleep apnea, and pathologic MRI findings other than mild white matter hyperintensities, as well as presence of dementia and/or parkinsonism fulfilling criteria for the diagnosis of PD, DLB, or MSA at baseline were excluded [17, 19-21]. Brain MRI was used to rule out brain diseases [17, 22]. The presence of white matter hyperintensities was not an exclusion criterion if the Wahlund scale was not > 1 for each brain region [23]. At baseline, all patients underwent the Movement Disorder Society Unified Parkinson Disease rating scale, motor section (MDS-UPDRS-III) to investigate the presence of parkinsonism, the Mini Mental State Examination (MMSE), as a measure of global cognitive functioning, as well as a comprehensive neuropsychological assessment (Seoul Neuropsychological Screening Battery [24]), including at least two tests for each of the main cognitive domains (verbal memory -Seoul Verbal Learning Test (SVLT) immediate recall, delayed recall, and recognition-, executive functions -TMT B and Controlled Oral Word Association Test (COWAT)-, attention and working memory -Trail-Making Test (TMT) A, digit span, and Color–Word Stroop Test (CWST)-, visuospatial abilities—Rey-Osterrieth Complex Figure Test (RCFT) copy- and language—Korean version of the Boston Naming Test (K-BNT)-) [17] to evaluate the presence of mild cognitive impairment (MCI) [25]. Thirteen patients phenoconverted to overt alpha-synucleinopathy at follow-up (7 PD, 5 DLB, 1 MSA; follow-up time: 35 ± 20.56 months; disease duration prior to diagnosis of iRBD 14.75 ± 16.04 months) while 27 patients were free from parkinsonism/dementia after a mean of 62 ± 29.49 months (disease duration prior to diagnosis of iRBD 24.95 ± 36.61 months). To note, follow-up time refers to survival time that indicates the interval (expressed in months) between the date of [^18^F]FDG-PET and the date of phenoconversion in converter patients, and between the date of [^18^F]FDG-PET and the last follow-up visit in non-converter patients. Demographic and clinical data are shown in Table 1.

**Table 1:** Demographic and clinical characteristics of Seoul iRBD patients. Values are shown as mean ± standard deviation [range]

	iRBD converted patients (*n* = 13)	iRBD non converted patients (*n* = 27)	*p*-value
Age (yr)	71 ± 4.85 [61–76]	69 ± 5.9 [62–80]	0.317
Education (yr)	9 ± 4.19 [2-16]	9 ± 5.39 [0–18]	0.953
Gender (M:F)	9:4	12:15	0.14
MMSE score	27 ± 2.28 [24-30]	27 ± 2.4 [23-30]	0.671
MDS-UPDRS-III score	8 ± 5.62 [2-17]	5 ± 4.43 [0–18.5]	0.101
Disease duration	14.75 ± 16.04 [3–60]	24.95 ± 35.61 [0.5–151]	0.332
Phenoconversion			
Survival Time (months)	35 ± 20.56 [12–84]	62 ± 29.49 [12–84]	**0.014**
PD	7 (53.84%)	/	
DLB	5 (38.46%)	/	
MSA	1 (7.69%)	/	

*Legend: DLB*, Dementia with Lewy Bodies; *F*, Female; *M*, Male; *MDS-UPDRS-III*, Movement Disorders Society-sponsored revision of the Unified Parkinson’s Disease Rating Scale, motor section; *MMSE*, Mini Mental State Examination; *MSA*, Multiple System Atrophy; *PD*, Parkinson’s Disease; *iRBD*, idiopathic REM sleep behaviour disorder; *REM*, Rapid eyes movements

*Note: Survival time indicates the interval (expressed in months) between the date of [*^*18*^*F]FDG-PET and the date of phenoconversion in converter patients, and between the date of [*^*18*^*F]FDG-PET and the last follow-up visit in non-converter patients*

Signifincant *p*-values are shown in bold

All subjects provided written informed consent before the enrolment in this study, in accordance with the Declaration of Helsinki.

### *[*^*18*^*F]FDG-PET*

[^18^F]FDG-PET was performed using a PET/computed tomography (CT) (Philips Gemini TF-64 PET/CT scanner, Philips Healthcare, Best, The Netherlands). All [^18^F]FDG-PET images were acquired in static mode and then subjected to affine and nonlinear spatial normalization into Montreal Neurological Institute (MNI) brain space using SPM12 (Wellcome Department of Cognitive Neurology, London, UK). All the default settings of SPM were used and a specific[^18^F]FDG-PET brain template was used as reference [26].

The spatially normalized set of images was then smoothed with a 10-mm isotropic Gaussian filter to account for individual anatomical variability and to improve the signal-to-noise ratio. Details of center specific imaging protocols and analysis have been described elsewhere [11, 15, 17, 27].

### Polysomnographic recording

Patients underwent overnight polysomnography (PSG). Sleep scoring was performed following the current criteria [28]. PSG derivations were placed according to recommended rules [28] to evaluate sleep features, respiratory, cardiac, and limb events. Patients were asked to withdraw melatonin, hypnotic medications, and antidepressant drugs for 2 weeks before the recording [16, 17]. Assessment was carried out by sleep medicine experts.

### iRBD conversion-related pattern (iRBDconvRP)

A previously derived and validated iRBDconvRP [15] was applied to [^18^F]FDG-PET scans of the forty iRBD patients enrolled to extract subject scores (z-transformed with respect to the non-converter iRBD patients) of the pattern expression, to be used in further analysis. Details of the derivation and validation of such pattern are described extensively elsewhere [15]. In brief, the iRBDconvRP was derived using an automated algorithm [29, 30] developed by the University Medical Center Groningen (UCMG), The Netherlands, based on the SSM-PCA method of Spetsieris and Eidelberg [31] implemented in Matlab (version 2020a; MathWorks, Natick, MA, USA). In brief, SSM-PCA allows to perform a spatial covariance analysis able to identify relevant pattern in [^18^F]FDG-PET data by taking into account the relationship between voxels across subjects. The degree of patterns expression is then reflected by single subject scores, so that disease activity is objectively assessed at single-subject level. Therefore, SSM-PCA was applied on a dataset of 30 converters and 46 non-converters iRBD patients, enrolled in two different Italian centers (Genoa and Rome Tor Vergata). Firstly, we derived two distinct patterns for each center, separately. Secondly, we pooled the data to identify the iRBDconvRP in the total dataset (Genoa + Rome Tor Vergata iRBD patients: 30 converters and 46 non-converters). The pattern analysis draws attention to the voxels that either co-vary positively or negatively. We could only speculate that both positive and negative components, from a pathologic perspective, represent higher and lower metabolism, respectively. Accordingly, the cerebellum, brainstem, anterior cingulate cortex, middle and mesial temporal and postcentral areas, and lentiform nucleus all have positive components of the iRBDconvRP (relatively higher glucose metabolism), while the posterior cingulate, precuneus, middle frontal gyrus, and parietal regions have negative components (relatively lower glucose metabolism).

Since the lack of an external group, we performed a leave-one-out cross validation (LOOCV) [9, 32] to confirm the stability of the pattern.

## Statistical analysis

Normal distribution of variables was checked using Shapiro–Wilk test. Continuous variables were compared either using unpaired *t*-test (normally distributed) or Wilcoxon-Mann–Whitney test (non-normally distributed).

To determine sensitivity and specificity of the pattern, a receiver operating curve (ROC) was plotted based on z-transformed subject scores. The cut-off that gave optimum sensitivity and specificity, calculated with the Youden Index method [33], was chosen as the empirical optimal cut-point. Next, Kaplan–Meier survival analysis was performed to estimate the predictive power of phenoconversion from iRBD to an overt alpha-synucleinopathy, using pattern expression values, categorized as below or above the optimal cut-point previously computed by the Youden Index method. The survival time was set as the interval (expressed in months) between the date of [^18^F]FDG-PET and the last follow-up visit in non-converter patients, and between the date of [^18^F]FDG-PET and the date of phenoconversion in converter patients. The hazard ratio (HR) was calculated with a Cox regression, using age, sex and education as covariates. Moreover, time-dependent ROC curves were calculated and the AUC of each timepoints were compared (one time point every 12 months, from month 24 until month 84).

We then performed the same analysis using as optimal cut-point the previously validated iRBDconvRP cut-off (0.6414) [15], to check its reproducibility in the independent group of patients.

Statistical threshold was set at 0.05 and *p*-values were reported corrected for multiple comparisons using Bonferroni approach. Analyses were performed using MatLab (version 2020a; MathWorks, Natick, MA, USA), R version 3.6.0 (R Foundation for Statistical Computing, Vienna, Austria) and Stata software (StataCorp. 2013. Stata Statistical Software: Release 13. College Station, TX, USA: StataCorp LP).

## Results

### iRBDconvRP phenoconversion prediction ability

**Fig. 1:**
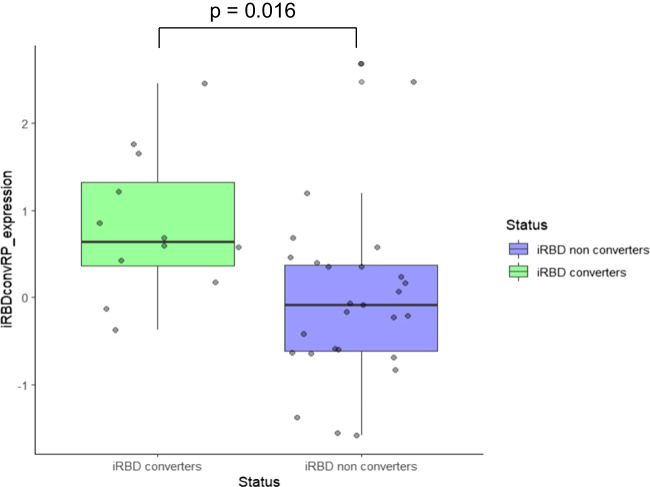
The plot represents the distribution of the iRBDconvRP expression (z-score) in Seoul iRBD patients. We found a greater expression of the iRBDconvRP patter in converters (green) patients, while a lower expression of the pattern was found in non-converters (blue) patients. This result confirms the discrimination ability of the pattern between converters and non-converters

Z-scores of iRBDconvRP expression were significantly higher in converters than non-converters (*p* = 0.016, empirical optimal cut-point = 0.411; Fig. 1).

**Fig. 2:**
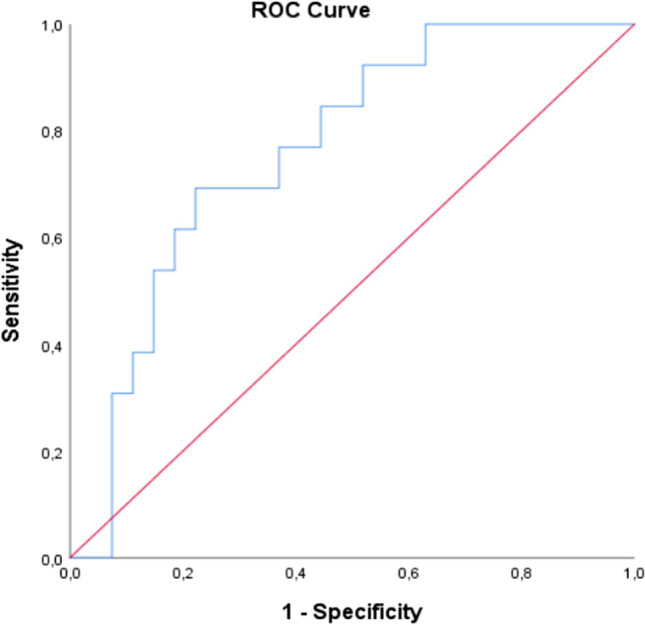
Results of the ROC analysis performed on individual z-scores showed a good sensitivity (69%) and specificity (78%) in discriminating converters and non-converters patients, with an AUC of 0.74. The empirical optimal cut-point was 0.411

**Fig. 3:**
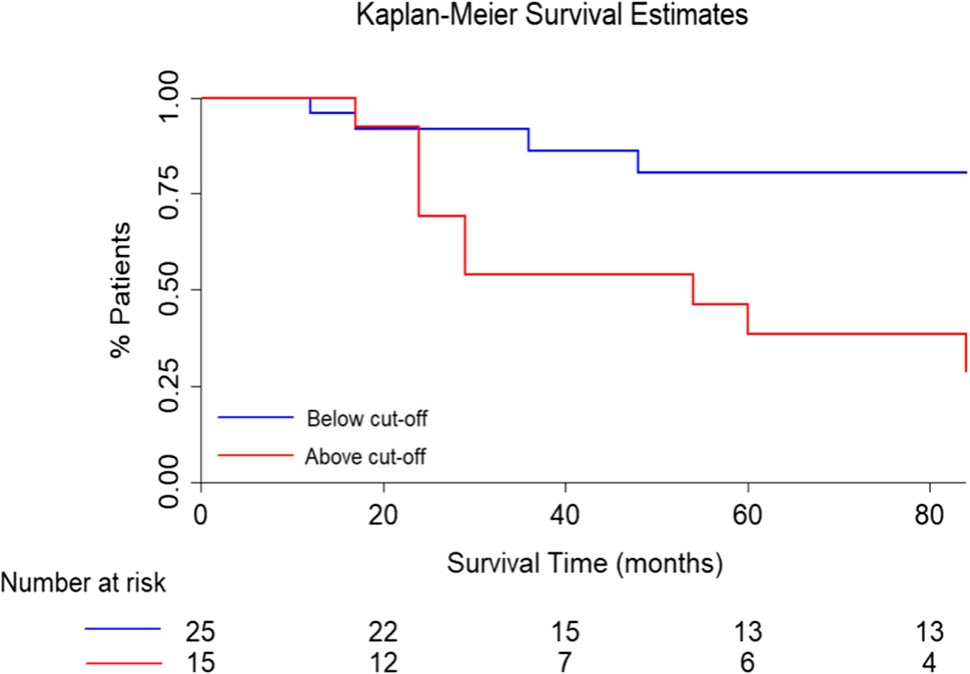
Results of the Survival analysis of the iRBDconvRP expression applied to Seoul iRBD patients. iRBD patients with iRBDconvRP expression lower than the empirical optimal cut-point found (0.411) are represented in blue, while iRBD patients with iRBDconvRP expression greater than the empirical optimal cut-point (0.411) are represented in red. The plot then shows that those patients with higher expression of the pattern tends to convert with higher probability at follow-up

At ROC analysis between z-scores of converters and non-converters, an area under the curve (AUC) of 0.74 was found (sensitivity: 69%, specificity: 78%, Fig. 2). Kaplan–Meier curves are reported in Fig. 3, the empirical optimal cut-off chosen as threshold is 0.411.

The prediction model was statistically significant (*p* = 0.012). On Cox-regression analysis, iRBDconvRP significantly predicted phenoconversion in iRBD patients (adjusted HR of 4.26, *p* = 0.027, C.I.95%: 1.18–15.39).

Time-dependent ROC analysis showed stable AUC throughout follow-up time (month 24: AUC 0.78; month 36: AUC 0.83; month 48: AUC 0.77; month 60: AUC 0.81; month 72: AUC 0.81; month 84: AUC 0.83). To note, after removing the MSA-converted patient (*n* = 1), the results did not change significantly.

When performing the analysis using the previously validated iRBDconvRP optimal empirical cut-off (0.6414), sensitivity decreased at 46%, while specificity increased at 85%. Cox regression analysis remained significant (*p* = 0.025), but the HR decreased at 3.36 (C.I.95%: 1.03–10.95).

## Discussion

In this study, we cross-validated a previously published brain metabolic pattern reflecting the risk of phenoconversion from iRBD to an overt alpha-synucleinopathy (iRBDconvRP) on an independent group of iRBD patients. Our results confirm the robustness and replicability of the iRBDconvRP.

It has been largely shown that neuroimaging biomarkers are essential for diagnosis and, in some cases, for predicting the clinical trajectory of different neurodegenerative diseases [34]. The identification of reliable biomarkers for phenoconversion is needed to plan robust clinical trials to apply in prodromal stages of neurodegenerative conditions, such as iRBD, which represents an ideal time window for disease-modifying therapies [35, 36].

The iRBDconvRP is a brain metabolic pattern directly related to the phenoconversion status in patients affected with iRBD, including clusters of relative hypermetabolism in the the cerebellum, brainstem, anterior cingulate cortex, middle and mesial temporal and postcentral areas, lentiform nucleus, as well as clusters of relative hypometabolism in the posterior cingulate, precuneus, middle frontal gyrus and parietal areas. The expression of the pattern itself, at the time of diagnosis, significantly predicted phenoconversion to an overt alpha-synucleinopathy at follow-up (either PD or DLB) with a high HR (7.42) [15]. Moreover, the iRBDconvRP showed a better prediction ability compared with that of a brain metabolic pattern derived from a group of de novo PD with evidence of RBD at baseline [15]. This may be due to the fact that the iRBDconvRP better represent the two main phenoconversion diagnosis (i.e. PD and DLB) than a more disease-specific pattern, such as the dnPDRBD-RP.

A biomarker is considered reliable and of clinical use when it is both replicable and applicable in other, independent groups. For example, the PDRP has been validated in several international cohorts [37, 38]. The importance of the PDRP as a biomarker of early stage PD was confirmed by Schindlbeck et al. (2020) [38], who reported that PDRP expression was abnormally increased in drug-naive, de novo PD patients recruited from an Italian center and an American one, also demonstrating the high reproducibility of the pattern across different cohorts. Similarly, Meles et al. (2020) [37] identified a PDRP in Italian, Dutch and Spanish patients and subsequently cross-validated them, finding a topographical overlap of the three patterns, confirming that PDRP is a universal feature of PD.

In the same way, here, we cross-validated the iRBDconvRP on an independent, external group of iRBD patients from the Seoul National University College of Medicine (South Korea), and we showed that the pattern was significantly more expressed in iRBD converter patients rather than non-converters. The iRBDconvRP also significantly predicted the phenoconversion to an overt stage of the disease, with a good HR (4.26).

Although, a lower HR was expected, since the iRBDconvRP was validated on an independent group rather than on the original derivation one.

Another factor that could have contributed to the lowering of the HR is the difference in the phenoconversion diagnosis. In fact, the iRBDconvRP was derived from a heterogeneous group of iRBD converter patients, mostly DLB (16 DLB vs. 14 PD), whereas in the external validation group the iRBD patients mostly phenoconverted to PD (7 PD, 5 DLB and 1 MSA). Lastly, the sample size of this replication group was relatively small. Even considering these factors that are expected when validating a biomarker on an independent sample of patients, the iRBDconvRP showed to be able to predict phenoconversion in an external, independent group of patients, without previous harmonization of the data. Moreover, the phenoconversion prediction ability of the iRBDconvRP remains high, and among the best published predictors [2, 14].

Of note, in the present study, 32.5% of iRBD phenoconverted to a full-blown alpha-synucleinopathy after a mean time of 35 months. This result well fits with recent literature [2, 3], although it is noteworthy that phenoconversion rates might be highly variable across different centers [39]. Furthermore, the phenoconversion time in the present study has been computed from [^18^F]FDG-PET and not from RBD diagnosis, thus the results are not directly comparable with other longitudinal studies that used RBD diagnosis as baseline.

Although this work has some limitations, firstly, the limited number of cases, however, the sample size of the present study is in line with the published cohorts of iRBD patients investigated by neuroimaging biomarkers. Second, it must be highlighted that the iRBDconvRP was derived in a Caucasian cohort of patients, while it was validated in the present study in an Asian cohort of patients. This may have contributed to lowering the prediction ability of the pattern, due to ethnic diversities between the two cohorts. On the other hand, this may also be considered a strength of the study because, despite the ethnic diversity, the iRBDconvRP still confirmed its ability in significantly predicting phenoconversion in iRBD patients. This will be of outmost importance for future clinical trials that would be conducted worldwide, including patients of different ethnicities.

In conclusion, in this study we showed the replicability of the iRBDconvRP as a biomarker of phenoconversion in iRBD patients as a prodromal stage of alpha-synucleinopathies. The importance of brain metabolic patterns has been proven to be particularly useful not only in the diagnostic process, but also in the prediction of clinical outcome in neurodegenerative diseases [11, 14, 37]. The iRBDconvRP showed promising results in identifying iRBD patients at high risk of short-term phenoconversion, but larger, multicentric studies are needed to further validate it.

## Data Availability

The data that support the findings of this study are available from J.Y.L upon reasonable request.
